# Current perspectives on China’s national essential medicine system: primary care provider and patient views

**DOI:** 10.1186/s12913-016-1283-z

**Published:** 2016-01-26

**Authors:** Yan Song, Ying Bian, Lingui Li

**Affiliations:** 1 Shandong Institute of Medicine and Health Information, Shandong Academy of Medical Sciences, 18877 Jingshi Lu, Jinan, Shandong Province China; 2 State Key Laboratory of Quality Research in Chinese Medicine, Institute of Chinese Medical Sciences, University of Macau, Avenida da Universidade, Taipa, Macau, China; 3 College of Management, Ningxia Medical University, 1160 Shengli Street, Yinchuan, Ningxia Province China

**Keywords:** Patient satisfaction, Essential medicines policy, Primary health care, China

## Abstract

**Background:**

The National Essential Medicine System (NEMS) is a new policy launched by the Chinese government in 2009. The effects of its introduction have been widely investigated. However, little research has focused on individual patients’ perspectives. The purpose of this study was to examine current understanding and opinions of China’s NEMS of primary care providers (PCPs) and patients.

**Methods:**

Providers (*n* = 134) and patients (*n* = 175) were examined based on self-completed questionnaire surveys conducted in 16 primary healthcare centers in Ningxia, northwestern China. Questions addressed the topics of: participants’ socio-demographic characteristics; awareness of NEMS policies; perceptions of NEMS-related changes; satisfactions with NEMS.

**Results:**

The patients had a low awareness of NEMS while a majority of providers were familiar with NEMS. All participants were satisfied with the price and quality of essential medicines, but not satisfied with the quantity. Patients felt there had been a decrease in their total medical expenses per visit and improved pharmaceutical services. Most providers perceived no change in their personal or departmental income. The overall satisfaction rate related to NEMS among providers and patients was 92.54 and 93.31 %, respectively. Overall there was a link between knowledge about NEMS and satisfaction with the program: patients who had greater knowledge of reimbursement policy, and the providers with greater knowledge of NEMS, reported higher satisfaction.

**Conclusions:**

The findings revealed a high level of satisfaction towards NEMS among primary care providers and patients, which is a reflection of the improvements in the health care system. However, some patients’ low awareness of NEMS should be paid attention to, as it could reduce their knowledge of essential medicines and hinder the full potential of NEMS.

**Electronic supplementary material:**

The online version of this article (doi:10.1186/s12913-016-1283-z) contains supplementary material, which is available to authorized users.

## Background

In response to poor access to medications and the high cost of drug therapy in developing countries, the World Health Assembly in 1975 introduced the concepts of “essential drugs” and “national drug policy”, and they quickly became part of the global public health vocabulary. Essential medicine has been regarded as the most cost-effective element of public health after immunization and key health promotion habits [1].

Following the resolution of World Health Assembly, the Chinese government embraced the concept of essential medicines in 1979 and produced the first edition of the national essential medicine list (EML) in 1982 [2]. Although the EML has existed for almost three decades in China, the list was excessive and only a few policies on essential medicines were in place. Until fairly recently China lacked a comprehensive national policy on essential medicines. Thus the EML had no or limited impact on China’s healthcare system and essential medicines did not realize their full potential [3].

Along with unsuccessful attempts to establish the essential medicine system in China, problems in the pharmaceutical sector have become major source of public criticisms. Drug expenditure per capita in real terms increased from 36.85 yuan in 1990 to 729.30 yuan in 2011 in China, an annual rate of increase of 15.3 %, well above overall economic growth [4, 5]. Chinese households devoted 40–60 % of their out-of-pocket healthcare expenditures to drugs between 1995 and 2008 according to the Chinese Health Statistical Digest [6]. The medically inappropriate and economically inefficient use of drugs also occurred frequently [7]. At least three out of ten patients were prescribed injections at community health institutions, which was two to three times higher than the WHO standard and the estimates from other developing countries between 2007 and 2009 [8]. The overuse of antibiotics is another common form of medicine abuse in China. China is one of the heaviest users of antibiotics, with 70 % of prescriptions containing antibiotics [9].

With increasing concerns regarding access to safe and effective essential medicines, in April 2009 the State Council of China launched the National Essential Medicine System (NEMS) with the goals of cutting the profit link between healthcare facilities, doctors, and drugs, and to improve drug availability, affordability, and rational use [10]. NEMS was initially designed for public primary healthcare facilities, with the intention of extending it to private providers and hospitals.

The newly built NEMS went beyond the scope of an essential medicines list but established a systematic process that involves formal appraisals, medicine delivery and distribution system, and rational usage. A centralized procurement system was developed for essential medicines to promote the merging and reorganization of pharmaceutical production and distribution companies. All government-owned primary healthcare centers were required to stock and prescribe drugs solely from the EML and to sell them at cost, a policy aimed at ending the interest link between physicians and drugs and reducing incentives for health care providers to over-prescribe. All essential medicines were included in the reimbursable medicine list, and had copayment rates lower than those of other medicines.

To date, the initial three-year implementation plan has been realized. The effects of its introduction in China have been widely investigated. However, little information is focused on people’s perspectives. Clearly, there is a need to investigate the patients and professionals’ view as they are the direct recipients and providers of essential medicines and this knowledge will aid in further improving the implementation of NEMS. Consequently, the purpose of this study was to examine patients’ and primary care providers’ current understanding of and opinions about China’s national essential medicine system, and to provide the baseline data for policy improvement.

## Methods

### Subjects

A questionnaire survey was conducted in Ningxia, China during December 2011. Ningxia is an autonomous region of China located in the northwest part of the country. It has the third smallest GDP in China and is representative of an undeveloped region. The feedback in such a place could be an important indicator for measuring the quality of the new NEMS.

Sixteen primary healthcare centers (PHCs) were randomly selected across Ningxia using stratified sampling in order to include areas with varying levels of socioeconomic status. Twenty subjects were surveyed in each PHC, including ten PCPs and ten patients.

The research methodology was approved by the ethics committee at the Institute of Chinese Medical Sciences at the University of Macau. The identities of all participants were kept confidential, and all were specifically informed that they were free to refuse to participate in the study or withdraw at any moment during the study. Written informed consent was obtained from the participants before their questionnaire survey.

### Instrument

Two separate self-completed questionnaires were supplied to providers and patients respectively in this study (see Additional files 1 and 2). Both questionnaires consisted of four parts. Questions addressed the topics of: socio-demographic characteristics; awareness of NEMS policies; perceptions of NEMS-related changes; satisfactions with NEMS. The responses for most questions were measured on a 5-point Likert Scale, with the lowest response on the one end of the scale and the highest response on the other end of the scale. General satisfaction regarding NEMS was evaluated on a 10-point scale, with one representing very unsatisfied and ten representing very satisfied. At the end of both questionnaires, an open-ended question was included to further investigate their demands of essential medicines.

### Data analysis

The questionnaires were coded, checked for accuracy and analyzed using IBM SPSS Statistics Version 19.0 (Armonk, New York, USA). Category data was analyzed for frequency distribution and percentage of subjects; quantitative data was analyzed by mean and standard deviation (SD) value. A multiple linear regression was employed to analyze the factors affecting satisfaction. The regression analyses were done by treating the general satisfaction score given by participants (1–10) as a dependent variable, and gender, age, education, income, knowledge of NEMS etc as independent variables. Table 1 showed the assignments of independent variables.

**Table 1:** Assignments of independent variables in regression analyses of satisfaction

Independent variable	Variable assignment
Gender^ab^	Male = 0; Female = 1
Age^ab^	<30 = 1; 30–50 = 2; >50 = 3
Education level^ab^	Primary school and below = 1; Middle school = 2; High school = 3; Junior college = 4; University and above = 5
Household income per month^a^	<1000 = 1; 1000–1999 = 2; 2000–2999 = 3; 3000–4999 = 4; 5000 and more = 5
Monthly income^b^	<1000 = 1; 1000–1999 = 2; 2000–2999 = 3; 3000–4999 = 4; 5000 and more = 5
Training times^b^	Actual value
Knowledge on NEMS^ab^	Have no idea = 1; Heard of = 2; Relative familiar = 3; Familiar = 4; Highly familiar = 5
Knowledge on EML^b^	Have no idea = 1; Heard of = 2; Relative familiar = 3; Familiar = 4; Highly familiar = 5
Knowledge on medicine zero-profit policy^a^	Have no idea = 1; Heard of = 2; Relative familiar = 3; Familiar = 4; Highly familiar = 5
Knowledge on medicine reimbursement policy^ab^	Have no idea = 1; Heard of = 2; Relative familiar = 3; Familiar = 4; Highly familiar = 5

Note: ^a^independent variables for patients’ satisfaction

^b^independent variables for professionals’ satisfaction

The general satisfaction scores were defined as follows: >8.5, absolutely satisfied; 6.5–8.5, quite satisfied; 4.5–6.5, relative satisfied; 2.5–4.5, dissatisfied; and <2.5, very dissatisfied. The final score for satisfaction was expressed as a rate and the formula was [8]: the overall satisfaction rate = (No. of absolutely satisfied + No. of quite satisfied + No. of relative satisfied)/ the total number of subjects*100 %.

## Results

### Demographic characteristics of the study participants

A total of 134 PCPs and 178 patients returned the questionnaires. Three patients failed to provide complete information, leaving a total of 309 valid sets of responses. The response rate was 96.56 %. Of the patients who responded, the mean age was 41.23 (SD = 13.18), with half being male (50.3 %). Most were with middle school or lower education (88.6 %). The majority of them (94.3 %) were insured by New Rural Cooperative Medical Scheme. Of the 134 providers, age range was 18–61 years (mean 35.43, SD = 8.93). By gender, female health care providers contributed 57.5 % with the male to female ratio of 0.8:1. Most were junior college graduates (64.2 %) and the mean length of employment was 12.4 (SD = 9.29) years. The profile of questionnaire respondents was summarized in Table 2.

**Table 2:** Demographic characteristic of study participants

	Patients		Providers	
Characteristics	N	%	N	%
Total	175	100	134	100
Gender				
Male	88	50.3	57	42.5
Female	87	49.7	77	57.5
Age (years)				
<30	32	18.3	30	22.4
30–50	99	56.6	94	70.1
>50	44	25.1	10	7.5
Education level				
High school and below	170	97.1	24	17.9
Junior college	3	1.7	86	64.2
Bachelor	2	1.1	24	17.9
Length of employment (years)				
<5	-	-	33	24.6
5–10	-	-	31	23.1
11–20	-	-	47	35.1
>20	-	-	23	17.2
Professional qualification				
NA	-	-	30	22.4
Primary	-	-	72	53.7
Junior	-	-	30	22.4
Senior	-	-	2	1.5
Working department				
Physicians	-	-	76	56.7
Pharmacists	-	-	19	14.2
Nursing staff	-	-	21	15.7
Health technicians	-	-	7	5.2
Administration staff	-	-	11	8.2
Monthly income (yuan)				
<1000	-	-	22	16.4
1000–2000	-	-	67	50
>2000	-	-	45	33.6
Household income per month (yuan)				
<1000	118	67.4	-	-
1000–2000	36	20.6	-	-
>2000	21	12	-	-

### The responses of patients

Almost 80 % patients did not know NEMS. Only 19 (10.9 %) showed familiar or relative familiar and nobody was quite familiar with NEMS. In addition, 134 (76.6 %) of the patients did not know essential medicines were sold at cost in PHCs. As for the reimbursement policy of essential medicines, 86 (49.1 %) were familiar or relative familiar, and 71 (40.6 %) still had no idea.

Eighty-two (46.8 %) of the 175 responded patients felt medicine prices had decreased or greatly decreased after NEMS implementation. 80 (45.7 %) thought there was no change and 13 (7.5 %) felt the prices had increased. Nevertheless most patients (86.3 %) were satisfied or very satisfied with the current price level. Only 21 (12.0 %) felt dissatisfied and 3 (1.7 %) were very dissatisfied. Furthermore, a total of 95 (54.3 %) patients thought that total medical expenses were reduced or greatly reduced after NEMS. However 11 (6.3 %) felt that total medical expenses had increased. In addition, many patients felt there was no quality loss and a total of 134 (76.6 %) patients were satisfied or very satisfied with the quality of essential medicines. But regarding the quantity of essential medicines, more than half (52.6 %) of them felt that the amount of medication did not meet their demands. The medicines they proposed to supplement include (listed by frequency of the occurrence): common cold medicines, gynecological medicines, cardiovascular medicines, nutrition medicines (i.e. healthcare products, vitamins, cod liver oil), anti-inflammatory medicines, paediatric medicines and more. Moreover, 116 (66.3 %) of patients felt there had been an improvement in pharmaceutical service at PHCs. A high proportion (96 %) of respondents felt satisfied or very satisfied with current medicine dispensing practices. As for professional services to help make the best use of medicines, the overall percentage of patients who were satisfied or very satisfied was 96.6 %.

The general satisfaction scored by the surveyed patients ranged between 2 and 10 with an average of 7.16 (SD = 1.86), indicating that the patients at PHCs were quite satisfied with NEMS. The overall satisfaction rate was 93.31 %.

### Primary care providers’ responses

PCP’s responses revealed that 131 (97.8 %) of the 134 providers were familiar or very familiar with the NEMS program. A majority of them (97.1 %) had an idea of EML. Specifically, 16 (11.9 %) felt very familiar; 72 (53.7 %) were familiar; and 41 (30.6 %) were relatively familiar. In the previous two years, 46.3 % of the PCPs received trainings on NEMS, although most of them received training only once or twice (Table 3). However, all participating PCPs expected to get more training.

**Table 3:** Frequency of trainings on NEMS to primary care providers

Training times	No. of respondents	Percent
0	72	53.7
1	23	17.2
2	20	14.9
3	7	5.2
≥4	12	8.8
Total	134	100

A total of 132 (98.5 %) PCPs were satisfied or very satisfied with the pricing of essential medicines. And 123 (91.8 %) were satisfied or very satisfied with the quality of essential medicines. Regarding the effects of essential medicines, 48 (35.8 %) of the respondents felt they were equivalent to those of non-essential medicines. But 41 (30.6 %) thought the effects of essential medicines tended to be worse. Furthermore, a majority of PCPs (81.4 %) felt that the amount of essential medicines did not meet their clinical needs. The medicines they proposed to supplement included (listed in order of frequency): gynecological medicines, cardiovascular medicines, paediatric medicines, digestive and stomachic medicines, emergency medicines, antibiotics, common cold medicines, rheumatism medicines, and so on. Following the implementation of NEMS, about 42 (31.3 %) PCPs thought their department income was reduced but most (66, 49.3 %) thought there was no difference. The majority of respondents (80.6 %) reported that there was no change in their personal incomes.

Close to 90 % of surveyed providers expected to promote NEMS to the whole country. The general satisfaction scored by PCPs ranged between 2 and 10 with an average of 6.93 (SD = 1.91), indicating that the providers who responded to this survey were quite satisfied with NEMS. The overall satisfaction rate was 92.54 %.

### Factors influencing satisfaction

In multi-variable linear regression analysis, the general satisfaction of patients was independently associated with their knowledge on reimbursement policy (*P* = 0.009, OR = 0.317, 95 % CI: 0.08–0.554). And the general satisfaction of providers was independently associated with their knowledge on NEMS (*P* = 0.03, OR = 0.663, 95 % CI: 0.234–1.092). For both of them, the more they knew the relevant information, the more they were satisfied with NEMS.

## Discussion

### Awareness

The findings of this study showed that patients in Ningxia had a low awareness of NEMS. Most of them only knew the policy name and did not know its specific content, let alone the significance of promoting NEMS. Other research in China also produced similar findings. For instance, a survey in Nanjing showed less than one fifth of patients had heard of NEMS and most had no awareness [11]. Patients in under-developed areas have poor access to medicines and should have been a target population of NEMS. However poor understanding of the importance of NEMS in improving access to and rational use of medicines might lead to low recognition of essential medicines and hinder the full potential of essential medicines. Thus, it is necessary to enhance the information available to patients through a variety of flexible ways. In Indonesia, a community poster competition raise awareness about a similar health program has proved extremely successful [12].

On the other hand, the findings indicated that PCPs had a certain awareness of NEMS. The majority of providers were familiar with NEMS and EML. Compared with an investigation in another western province where only 63.7 % were reported to have an idea of NEMS [13], the PCPs in Ningxia had a high awareness. The results from this research also indicated that there is still a lack of normal and systematic training for PCPs.

### Perception and satisfaction

The satisfaction among patients and providers in this study was mainly understood from their perceptions of the price, quality and quantity of essential medicines. Furthermore, a close eye was kept on patients’ perceptions of total medical expenses and pharmaceutical services at PHCs. Also, providers’ views about changes to their personal and departmental incomes were taken into account. This is because drug revenue was previously the primary source of income for PHCs in China (ranging from 50 to 90 % of total revenue). Thus the implementation of a zero-profit pharmaceuticals policy could cause a substantial financial loss for PHCs, resulting in shortages for routine operational activities [14].

First, a majority of patients and providers were satisfied with the price and the quality of essential medicines. Most patients perceived that drug prices had decreased. This finding reinforced the findings of a Chinese Ministry of Health report, that “drug prices in PHCs went down about 30 % in 2010” [15]. However both patients and providers believed that the quantity of essential medicines did not meet their demands, which is consistent with several previous studies in China [16, 17]. Many factors contribute to unmet demand for medicines. The poor selection of essential medicines might be a dominant contributor. A study in three rural counties in western China reported that about 1/3 of the drugs in common use by local people are not on the list, and 1/3 of drugs on the list were rarely used [16]. The medicine use habit was another important factor. Following the implementation of the NEMS, all PHCs were required to stock and prescribe drugs solely from the EML. Neither physicians nor patients have adapted to this new medication distribution system. Delivery efficiency could also be a contributor. The low profit margins for essential medicines may discourage distributors from guaranteeing their supply. In addition, all provinces have now increased the number of essential medicines in their local supplementary list. Ningxia added 64 additional medicines (37 traditional medicines and 27 chemical and biological medicines), which is the lowest number of essential medicines listed in China at the provincial level. This is also a reason that providers and patients in Ningxia are not satisfied with the quantity of essential medicines [18]. Responses from both groups suggested adding more gynecological medicines, cardiovascular medicines, paediatric medicines, antibiotics and common cold medicines to the EML.

Next, the findings indicated that patients perceived that there had been a decrease in their total medical expenses. The decreases in drug prices and more rational prescribing behaviors might be major contributors. In many developing countries, medicines represent the largest household health expenditure. By focusing pharmaceutical expenditure on essential drugs, the cost-effectiveness of government and out-of-pocket drug expenditure can be enhanced, and positive health impacts heightened [19]. In the meanwhile, most PCPs did not feel that there had been a change in their personal or departmental income. This result might imply that PHCs in Ningxia were well compensated. However, one third of the PCPs still perceived that their departmental incomes had decreased. The PHCs would be dealing with greater numbers of patients in the post-implementation period of NEMS. It is essential to ensure sustainable financial compensation for PHCs to support their routine operational activities and a high quality of health services.

Finally, the results indicated that NEMS was welcomed by both patients and providers. A research project conducted by Renmin University’s Health Reform and Development Center in Shijiazhuang and Beijing also confirmed this finding [3]. Moreover, patients with better knowledge about reimbursement policy and PCPs with better knowledge of NEMS showed a higher level of satisfaction regarding NEMS. This finding also supports to the implications of Knowledge-Attitude-Practice (KAP) theory. Providing sufficient information about NEMS can increase patient and PCP satisfaction with essential medicines, thereby reducing the demands for the drugs which will not bring additional health benefits. Also, this finding indicated that patients were more concerned with out-of-pocket expenditures regarding essential medicine reform.

### Limitations

Findings presented in this study should be considered in light of some limitations. First, the samples were limited to the subjects in Ningxia. Generalization from the findings should be treated with caution. Second, the use of a self-administered questionnaire is known to have limitations, as participants might have difficulties in understanding and responding to some questions. Likewise participants may not respond to all questions. Third, our sample size was relatively small, which might reduce the external validity of the results. However, Ningxia was a pioneer of China’s NEMS as well as a representative of under-developed areas. Despite these limitations, we believe our findings could provide a valuable source of information for policy makers and also be useful to developing nations in advocating for the incorporation of effective NEMS.

## Conclusions

This study demonstrates a high level of satisfaction with China’s NEMS among primary care providers and patients, which is a reflection of improvements in the health system. However low awareness about NEMS policies among patients should be paid attention to, as it may weaken their recognition of essential medicines and hinder the policy’s implementation. Thus, consideration should be given to future public education programs. In addition, satisfaction with NEMS could be significantly increased by improving the selection of medicines, when the distribution system is responsive and efficient and the compensation for PHCs is sufficient and sustainable.

## Additional files

Additional file 1: **S1: Questionnaire for patients.** (DOCX 32 kb)

Additional file 2: **S2: Questionnaire for primary care providers.** (DOCX 31 kb)

## Abbreviations

EML: Essential Drug List
NEMS: National Essential Medicine System
PCP: Primary care provider
PHC: Primary healthcare center
SD: Standard deviation
WHO: World Health Organization
