# Supplementary material for: Current perspectives on China’s national essential medicine system: primary care provider and patient views
Source: BMC Health Serv Res. 2016 Jan 26;16:30. doi: 10.1186/s12913-016-1283-z (PMC4729152; doi:10.1186/s12913-016-1283-z)
Supplement: Supplementary file 1 — S1: Questionnaire for patients. (DOCX 32 kb) [file 12913_2016_1283_MOESM1_ESM.docx]

**S1: Questionnaire for patients**

**Section I. Socio-demographic characteristics**

1. Gender: 口Male 口Female
2. Date of birth: ____(month)/____(year)
3. Your highest level of education:

口University and above 口Junior college 口High school and below

1. The average income of your household (yuan per month):

口Less than1000 口1000-2000 口More than 2000

1. Have you participated in New Rural Cooperative Medical Scheme?

口Yes 口No

**Section II. Awareness of NEMS policies**

(Please check the box that corresponds with your level of awareness.)

1. Do you know the National Essential Medicine System (NEMS)?

口Have no idea 口Heard of 口Relative familiar 口Familiar 口Quite familiar

1. Do you know essential medicines are sold at cost in primary healthcare centers?

口Have no idea 口Heard of 口Relative familiar 口Familiar 口Quite familiar

1. Do you know the reimbursement policy of essential medicines?

口Have no idea 口Heard of 口Relative familiar 口Familiar 口Quite familiar

**Section III. Perceptions of NEMS-related changes**

(Please check the box that corresponds with your agreement.)

1. Have you felt medicine prices decreased after NEMS implementation in primary healthcare centers?

口Greatly increased口Increased 口Neutral 口Decreased 口Greatly decreased

1. Have you felt total medical expenses decreased after NEMS implementation in primary healthcare centers?

口Greatly increased口Increased 口Neutral 口Decreased 口Greatly decreased

1. Do you think the quantity of essential medicines can satisfy your demands?

口Strongly disagree口Disagree 口Neutral 口Agree 口Strongly agree

1. Do you think the pharmaceutical service at primary healthcare centers improved after NEMS?

口Strongly disagree口Disagree 口Neutral 口Agree 口Strongly agree

**Section IV. Satisfactions with NEMS**

(Please check the box that corresponds with your level of satisfactions.)

1. Are you satisfied with the current price level of medicines?

口Very unsatisfied口Unsatisfied 口Neutral 口Satisfied 口Very satisfied

1. Are you satisfied with the quality of essential medicines?

口Very unsatisfied口Unsatisfied 口Neutral 口Satisfied 口Very satisfied

1. Are you satisfied with current medicine dispensing practices?

口Very unsatisfied口Unsatisfied 口Neutral 口Satisfied 口Very satisfied

1. Are you satisfied with current professional services to help make the best use of medicines?

口Very unsatisfied口Unsatisfied 口Neutral 口Satisfied 口Very satisfied

1. What is your general satisfaction regarding NEMS? (0-10 points)

Very unsatisfied Very satisfied

1. 2 3 4 5 6 7 8 9 10

* Please provide any additional medicines that you want to supplement to current essential medicine list.

_____________________________________________________

_____________________________________________________
