# Supplementary material for: Current perspectives on China’s national essential medicine system: primary care provider and patient views
Source: BMC Health Serv Res. 2016 Jan 26;16:30. doi: 10.1186/s12913-016-1283-z (PMC4729152; doi:10.1186/s12913-016-1283-z)
Supplement: Supplementary file 2 — S2: Questionnaire for primary care providers. (DOCX 31 kb) [file 12913_2016_1283_MOESM2_ESM.docx]

**S2: Questionnaire for primary care providers**

**Section I. Socio-demographic characteristics**

1. Gender: 口Male 口Female
2. Date of birth: ____(month)/____(year)
3. Your highest level of education:

口University and above 口Junior college 口High school and below

1. The length of employment: ___year(s)
2. Your professional qualification: 口NA 口Primary 口Junior 口Senior
3. Your working department:

口Physicians 口Pharmacists 口Nursing staff 口Health technicians 口Administration staff

1. Your average income per month (yuan):

口Less than1000 口1000-2000 口More than 2000

**Section II. Awareness of NEMS policies**

(Please check the box that corresponds with your level of awareness.)

1. Do you know the National Essential Medicine System (NEMS)?

口Have no idea 口Heard of 口Relative familiar 口Familiar 口Quite familiar

1. Do you know the essential medicine list?

口Have no idea 口Heard of 口Relative familiar 口Familiar 口Quite familiar

1. Have you received trainings on NEMS during the past two years?

口Yes (if so, how many times?___) 口No

1. Do you want to get more training?

口Yes 口No

**Section III. Perceptions of NEMS-related changes**

(Please check the box that corresponds with your agreement.)

1. Do you think the effects of essential medicines are equivalent to those of non-essential medicines?

口Strongly disagree口 Disagree 口Neutral 口Agree 口Strongly agree

1. Do you think the quantity of essential medicines can satisfy the clinical needs?

口Strongly disagree口 Disagree 口Neutral 口Agree 口Strongly agree

1. Have you felt your department income decreased following the implementation of NEMS?

口Greatly decreased口 Decreased 口Neutral 口Increased 口Greatly increased

1. Have you felt your personal income decreased following the implementation of NEMS?

口Greatly decreased口 Decreased 口Neutral 口Increased 口Greatly increased

1. Do you expect to promote NEMS to the whole country?

口Strongly disagree口 Disagree 口Neutral 口Agree 口Strongly agree

**Section IV. Satisfactions with NEMS**

(Please check the box that corresponds with your level of satisfactions.)

1. Are you satisfied with the pricing of essential medicines?

口Very unsatisfied口 Unsatisfied 口Neutral 口Satisfied 口Very satisfied

1. Are you satisfied with the quality of essential medicines?

口Very unsatisfied口 Unsatisfied 口Neutral 口Satisfied 口Very satisfied

1. What is your general satisfaction regarding NEMS? (0-10 points)

Very unsatisfied Very satisfied

1 2 3 4 5 6 7 8 9 10

*Please provide any additional medicines that you want to supplement to current essential medicine list.

_____________________________________________________

_____________________________________________________
